# Supplementary material for: Diversity of the Cnaphalocrocis medinalis gut bacterial community and its contribution to reproduction
Source: Microbiol Spectr. 2026 Apr 16;14(6):e03878-25. doi: 10.1128/spectrum.03878-25 (PMC13227977; doi:10.1128/spectrum.03878-25)
Supplement: Supplementary material — Supplemental methods; Fig. S1 to S3; Table S1. [file spectrum.03878-25-s0001.docx]

**Supplementary Material**

**Diversity of the *Cnaphalocrocis medinalis* gut bacterial community and its contribution to reproduction**

Zhichao Yao ^1^, Chengrui Xue^1^, Yang Ang^1^, Ziwei Wu^1^, Xin Liu^1^, Ying Liu^1^, Fang Liu^1#^, Qinjian Pan^2#^

^1^ College of Plant Protection, Yangzhou University, Yangzhou, China.

^2^ Joint International Research Laboratory of Agriculture and Agri-Product Safety, the Ministry of Education of China, Institutes of Agricultural Science and Technology Development, Yangzhou University, Yangzhou, China.

* Fang Liu ✉e-mail: liufang@yzu.edu.cn

* Qinjian Pan ✉e-mail: qjpan@yzu.edu.cn

**Supplementary Methods**

**Assessment of moth fertility, and quantification of food uptake in *C. medinalis* adults and larvae**

To evaluate fertility (hatching rate), moths were either treated with antibiotics (ABX group) or left untreated (conventionally-treated, CONV group) for 2 d. A single female-male pair was then transferred to a self-designed egg-counting device. The moths were provided with cotton balls soaked in 2.5 10% HS containing antibiotics for 3 d or with sterile HS as a control. Eggs laid on the plastic film at 4-5 d post emergence were collected and monitored for hatching. Fertility was calculated as the percentage of hatched larvae relative to the total number of eggs.

To quantify food uptake in *C. medinalis* adults, moths from the CONV and ABX-treated groups were allowed to feed on 2.5% or 10% HS supplemented with a final concentration of 0.32% FD&C Blue Dye #1 (catalog no. 861146, Sigma-Aldrich) for 4 h. After feeding, four moths per group were randomly selected, and their entire gut tissues were dissected and collected. The gut samples were emulsified in 1 ml of PBS using a homogenizer at 70 Hz/s for 120 s, followed by centrifugation at 12,000 × g for 20 min. Next, a 200 µl aliquot of the supernatant was loaded into a 96-well crystal plate, and the OD_633_ was measured with a Tecan Infinite^®^ M200 microplate reader (Tecan, Switzerland) to quantify the relative amount of dye ingested. For quantification of food uptake of larvae, newly-hatched larvae from CONV and ABX-treated groups were raised on wheat seedlings until they reached the 3^rd^ instar stage. Individual 3^rd^ instar larvae were then transferred to isolated wheat seedlings that were enclosed with mesh netting to prevent escape. After 24 h of feeding, leaves were photographed, and the consumed leaf area was quantified using ImageJ software.

**Table S1. Primers used for bacterial detection.**

| Primers | Sequence | Reference and target |
| --- | --- | --- |
| 338F | 5′-ACTCCTACGGGAGGCAGCAG-3′ | Huse et al. 2008/Masoud et al. 2011; for V3-V4 segments amplification |
| 806R | 5′-GGACTACHVGGGTWTCTAAT-3′ |  |
| 27F | 5′-GTTTGATCCTGGCTCAG-3′ | Lane DJ. 1991; for nearly full-length 16S rRNA gene amplification |
| 1492R | 5′-GGTTACCTTGTTACGACTT-3′ |  |
| Uni331F | 5′-TCCTACGGGAGGCAGCAGT-3′ | Bartosch et al. 2004; for detection of total bacteria population |
| Uni797R | 5′-GGACTACCAGGGTATCTATCCTGTT-3′ |  |
| hpa287F | 5′-GGCGCGCCATGATCTCY-3′ | Jordan et al. 2025; for detection of *Enterobacter* strains |
| hpa625R | 5′-ATGGTCGACCGCTGYATGTC-3′ |  |
| Actin QF | 5′-AAGGACCTGTACGCCAACAC-3′ | This study; for detection of *C. medinalis actin* in samples |
| Actin QR | 5′-GAGGGCGGTGATTTCCTTCT-3′ |  |

**Supplementary Figures**


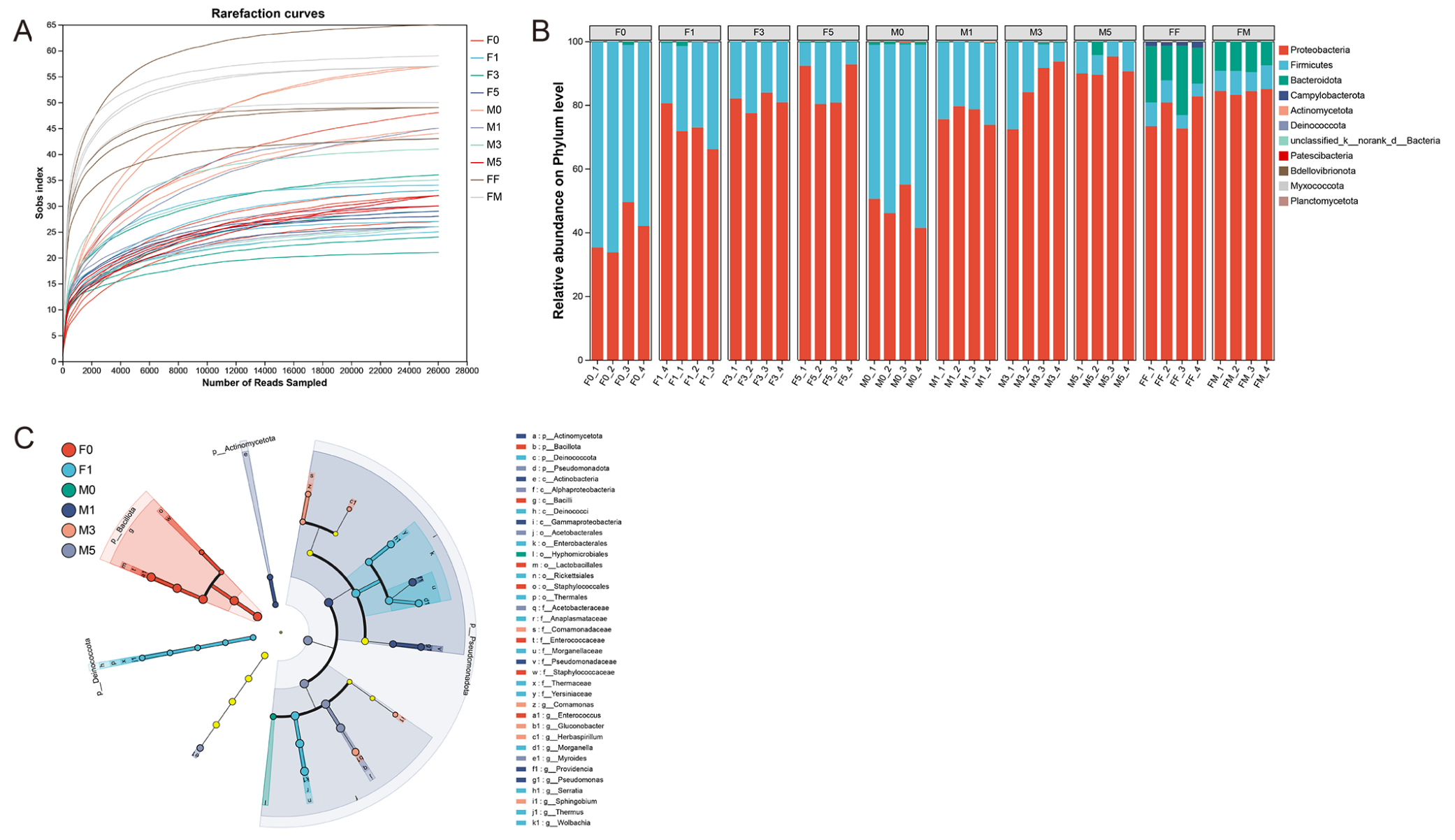


**Fig. S1 Analysis of sequencing depth and taxonomic composition of gut microbiota.** (A) Rarefaction curves show sequencing depth and saturation. (B) Stacked bar plots showing relative abundance of bacterial communities at the phylum level in laboratory and field *C. medinalis* adults. (C) Cladogram illustrating taxonomic differences among samples from different development stages, as identified by linear discriminant analysis effect size (LEfSe) (*P* < 0.05). Abbreviations: F0, F1, F3, and F5 represent females at 0, 1, 3, and 5 d post emergence; M0, M1, M3, and M5 show data for males at 0, 1, 3, and 5 d post emergence; FF and FM5 represent field females and field males, respectively.


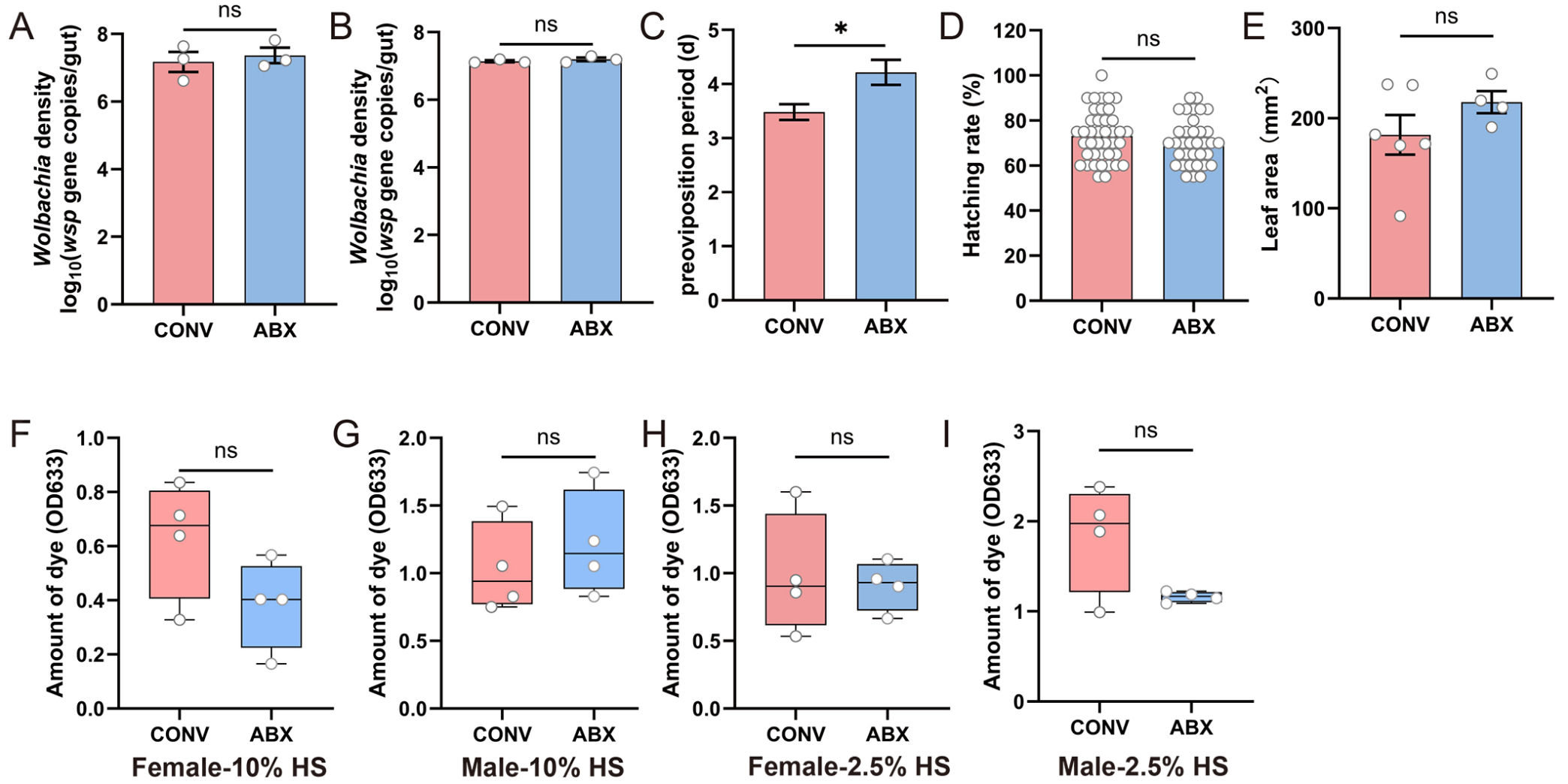


**Fig. S2 Effects of gut symbiont depletion on *Wolbachia* density, reproductive performance and feeding behavior in *C. medinalis*.** (A-B) Effects of ABX treatment on *Wolbachia* density in the gut of female *C. medinalis* fed with (A) 10% HS or (B) 2.5% HS, respectively. (C-E) Impact of gut bacterial elimination (ABX treatment) on the (C) preoviposition period, (D) egg hatching rate, and (E) feeding quantity of offspring larvae. (F-I) Effects of gut bacterial elimination on amount of food ingested by females and males fed with (F, G) 10% HS or (H, I) 2.5% HS, respectively. Food uptake in *C. medinalis* was measured by allowing adult moths from the CONV- and ABX-treated groups to feed on 2.5% or 10% HS supplemented with 0.32% FD&C Blue Dye #1 for 4 h. The asterisks indicate significant differences (**P* < 0.05; ns, not significant). Abbreviations: ABX, antibiotic treatment; CONV, conventionally-reared; HS, honey solution.


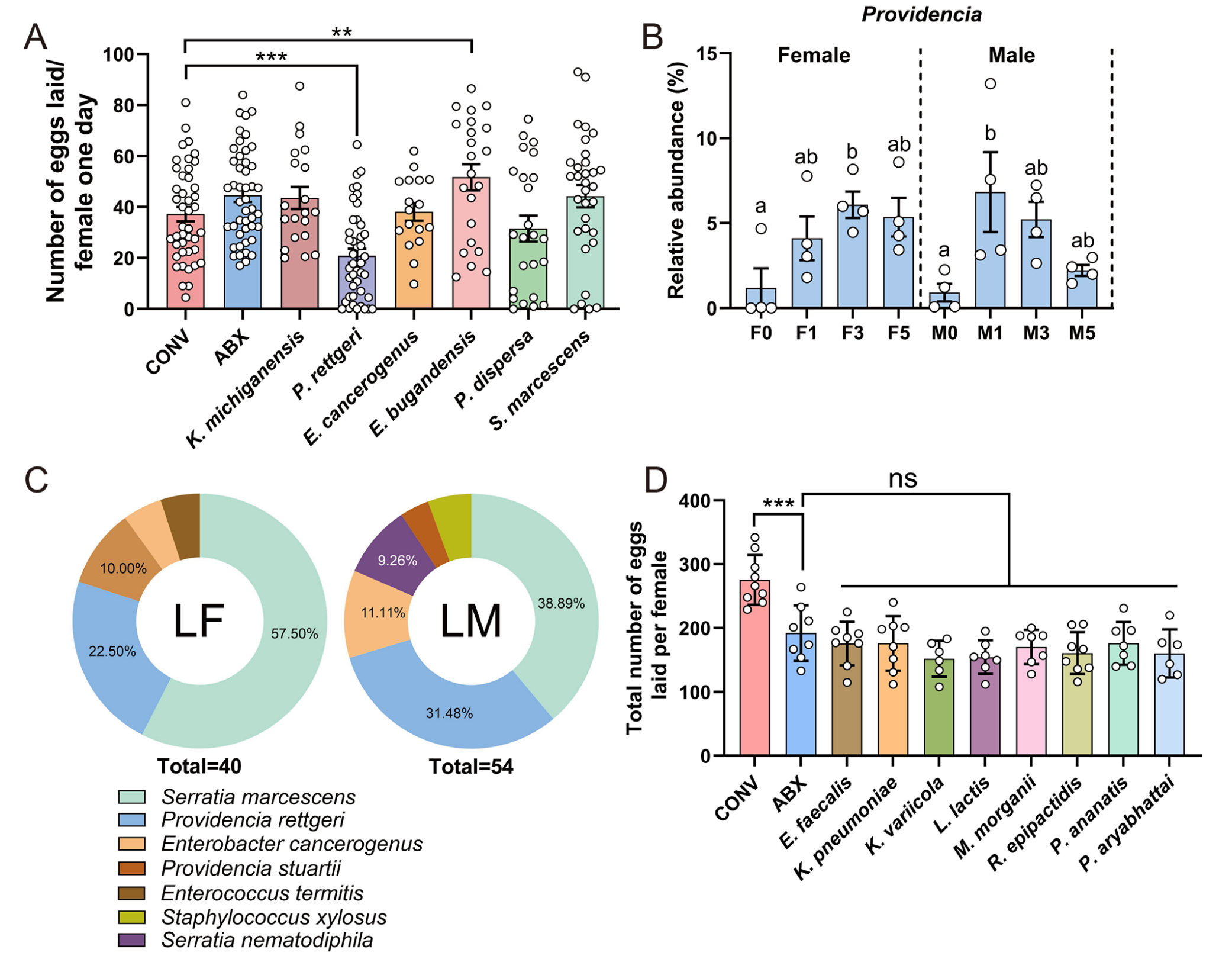


**Fig. S3 Effects of gut symbionts on oviposition in *C. medinalis* females.** (A) Peak number of eggs laid by females monoassociated with gut bacterial species isolated from laboratory populations supplied with 10% HS. (B) Dynamic change in the relative abundance of *Providencia* spp. across four adult age groups. (C) The composition of culturable bacteria in laboratory female and male adults at 7 d post-eclosion. (D) Total number of eggs laid by females monoassociated with gut bacteria isolated from field populations and reared with 2.5% HS. Asterisks indicate significant differences as follows: (***P* < 0.01; ****P* < 0.001; ns, not significant. Abbreviations: ABX, antibiotic treatment; CONV, conventionally-reared; HS, honey solution; LF, laboratory females; and LM, laboratory males. F0, F1, F3, and F5 represent females at 0, 1, 3, and 5 d post emergence; M0, M1, M3, and M5 show data for males at 0, 1, 3, and 5 d post emergence.

**Reference**

Bartosch S, Fite A, Macfarlane GT, McMurdo ME. 2004. Characterization of bacterial communities in feces from healthy elderly volunteers and hospitalized elderly patients by using real-time PCR and effects of antibiotic treatment on the fecal microbiota. Appl Environ Microbiol 70:3575-81. <https://doi.org/10.1128/AEM.70.6.3575-3581.2004>

Huse SM, Huber JA, Morrison HG, Sogin ML, Welch DM. 2008. Accuracy and quality of massively parallel DNA pyrosequencing. Genome Biol 8:R143. <https://doi.org/10.1186/gb-2007-8-7-r143>

Jordan S, Pothier JF, De Maayer P, Broders K, Kvitko BH, Coutinho TA, Smits TH. 2025. Design of genus-specific semi-nested primers for simple and accurate identification of *Enterobacter* strains. BMC Microbiol 25:456. https://doi.org/10.1186/s12866-025-04175-1Liu C, Zhao D, Ma W, Guo Y, Wang A, Wang Q, Lee DJ. 2016. Denitrifying sulfide removal process on high-salinity wastewaters in the presence of *Halomonas* sp. Appl Microbiol Biotechnol 100:1421-1426. <https://doi.org/10.1007/s00253-015-7039-6>

Lane, DJ. 1991. 16S/23S rRNA sequencing, p. 115-175. *In* E. Stackebrandt and M. Goodfellow (ed.), Nucleic acid techniques in bacterial systematics. John Wiley and Sons, New York, N.Y.

Masoud W, Takamiya M, Vogensen FK, Lillevang S, Al-Soud WA, Sørensen SJ, Jakobsen M. 2011. Characterization of bacterial populations in Danish raw milk cheeses made with different starter cultures by denaturating gradient gel electrophoresis and pyrosequencing. Int Dairy J 21:142-148. <https://doi.org/10.1016/j.idairyj.2010.10.007>
